# Valorization of kidney bean (*Phaseolus vulgaris* L.) pod powder: Multifactorial optimization of gluten‐free cake

**DOI:** 10.1002/fsn3.3813

**Published:** 2023-11-21

**Authors:** Ceyda Dadalı

**Affiliations:** ^1^ Food Engineering Department, Engineering Faculty Ege University İzmir Turkey

**Keywords:** gluten‐free cake, kidney bean pod, multifactorial optimization, *Phaseolus vulgaris* L.

## Abstract

The lifelong gluten‐free diet of celiac patients and gluten‐intolerant people prevents their balanced diet mainly due to starch‐rich products. The aim of this study is to determine optimum gluten‐free cake formulation having kidney bean (*Phaseolus vulgaris* L.) pod powder as fat replacer of up to 50% and rice flour replacer of up to 30% using multifactorial optimization approach. Central composite design was used to determine optimum formulation. The use of kidney beans in gluten‐free cake increased moisture, hardness, chewiness, L*, a*, b*, antioxidant activity, total phenolic content, and sensory evaluation scores (*p* < .05). The optimum gluten‐free cake is rich in protein (5.89%), phenolic compounds (0.51 mg GAE/g), antioxidant activity (1.93 μmol TE/g), and total dietary fiber (4.43%) with improved sensory properties. The optimum gluten‐free cake formulation prepared with kidney bean pod powder of 27.88% fat and 13.52% rice flour replacer provides higher specific volume, springiness, total phenolic content, antioxidant activity, and sensory analysis scores, and lower hardness and chewiness conditions. Gluten‐free cake containing kidney bean pod powder as fat and rice flour replacer at optimum ratio is a new healthier alternative with reduced fat content and improved nutritional and sensory properties for celiac patients and gluten‐intolerant people.

## Graphical abstract

The optimum usage ratio of kidney bean pods as fat and rice flour replacer in gluten‐free cake was investigated and it was determined that kidney bean pods can be used as 27.88% fat and 13.52% rice flour replacer under optimum conditions. Gluten‐free cake containing kidney bean pods as a fat and rice flour replacer can be a new alternative with improved nutritional and sensory properties for people on a gluten‐free diet and celiac patients.

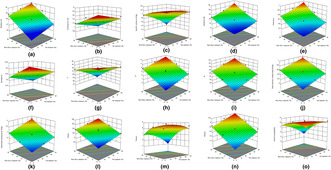

## 1 INTRODUCTION

Celiac disease is an autoimmune small intestine disease associated with malabsorption of nutrients in the presence of gluten found in wheat, rye, and barley (Azari et al., 2020). The prevalence of celiac disease is approximately 1% worldwide and increasing over time with female predominance (Caio et al., 2019; Segura et al., 2021). Celiac disease can occur at any age and has varied symptoms/manifestations (Caio et al., 2019). Symptoms of celiac disease are malnutrition, diarrhea, growth retardation, anemia, and fatigue (Xu et al., 2020). The gluten intolerance of celiac patients continues lifelong and the only way to treatment is gluten‐free diet (Azari et al., 2020). In order to produce gluten‐free food products, rice flour and starches are used instead of gluten‐containing ingredients such as wheat flour in the food formulation. For this reason, gluten‐free products have high‐starch content and low‐nutrient and ‐functional compound content (Arslan et al., 2019). At the same time, dietary fiber intake of celiac patients decreases with the consumption of starch‐rich food products (Brigagão et al., 2021). The diet of celiac patients with starch‐rich foods prevents them from having a balanced diet. In recent years, functional compounds have been added to gluten‐free products, contributing to the healthy diet of celiac patients (Yeşilkanat & Savlak, 2021).

The food industry that processes fruit and vegetables generates large amounts of by‐products and waste such as pods, peels, and seeds (Brigagão et al., 2021; de Sousa Sabino et al., 2020). Improper disposal of these waste and by‐products creates significant environmental problems (Larrosa & Otero, 2021). On the other hand, these fruit and vegetable by‐products have high nutrient and bioactive content (Brigagão et al., 2021). Therefore, these by‐products have the potential to be converted into pharmaceuticals and food products (Larrosa & Otero, 2021).

Cakes are sweet and baked snack products consumed by all levels of society due to ready‐to‐eat nature (Ammar et al., 2021). Cakes are cereal products generally produced using wheat flour. In order to produce gluten‐free cakes, rice flour and starch‐rich ingredients are added to the formulation to replace wheat flour. Various food by‐products are used in product formulations to improve the nutritional content of starch‐rich gluten‐free cakes and to increase the content of dietary fiber and functional compounds. In previous studies, by‐products namely, apple, orange, and carrot pomace, pomegranate seed, pineapple peel, banana peel, pumpkin seed, and green banana peel were used in gluten‐free cakes for enrichment (Azari et al., 2020; Brigagão et al., 2021; Kırbaş et al., 2019; Saeidi et al., 2018; Türker et al., 2016). Kidney bean (*Phaseolus vulgaris* L.) is an important legume crop cultivated and consumed worldwide (Hu et al., 2015). Due to the widespread consumption of kidney bean seeds, kidney bean pods are formed as a by‐product. To the best of our knowledge, there has been no study about multifactorial optimization of gluten‐free cake having kidney bean pod powder as fat and rice flour replacer. In this study, it was aimed to use kidney bean pod powder as fat and rice flour replacer in gluten‐free cake to improve the nutrient content and to determine the optimum gluten‐free cake formulation with a multifactorial optimization approach.

## 2 MATERIALS AND METHODS

### 2.1 Materials

Rice flour, sunflower oil, milk, egg, sugar, and baking powder were purchased from a local market in İzmir Turkey. Kidney bean (*Phaseolus vulgaris* L. var. Pinto) pods were obtained from producer in İzmir, Turkey, and prepared for drying within 24 h. All chemicals at analytical grade were supplied by Merck.

### 2.2 Experimental design

Face‐centered central composite design (CCD) with two numerical factors at three coded levels was generated using Design Expert version 11.0. Fat replacer and rice flour replacer ratio were defined as independent variables. These two independent variables, upper and lower levels, are shown in Table 1. Kidney bean pod powder was used as fat replacer of up to 50% and rice flour replacer of up to 30%. Moisture content, cooking loss, specific volume, hardness, chewiness, springiness, color values (L*, a*, and b*), and sensory analysis results (color, texture, flavor, and overall acceptability) were defined as responses to optimize the use of kidney bean pod powder as fat replacer and rice flour replacer in gluten‐free cake. The model equation derived for the response value prediction is given in Equation (1). In this equation, *R* response value, *β*_0_ constant, *β*_1_ and *β*_2_ linear, *β*_11_ and *β*_22_ quadratic and *β*_12_ interaction constants, and *X*_1_ and *X*_2_ values were independent variables.

(1) $$R=\beta_{1}X_{1}+\beta_{2}X_{2}+\beta_{11}X_{1}^{2}+\beta_{22}X_{2}^{2}+\beta_{12}X_{1}X_{2}$$

**TABLE 1:** Experimental conditions of face‐centered central composite design for fat replacer and rice flour replacer and gluten‐free cake ingredients.

Experiment no.	Fat replacer (%)	Rice flour replacer (%)	Sunflower oil	Kidney bean pod powder as fat replacer (g)	Rice flour (g)	Kidney bean pod powder as rice flour replacer (g)	Milk (g)	Egg (g)	Sugar (g)	Baking powder (g)
1	25	15	42.19	14.06	127.50	22.50	112.50	93.75	168.75	5.63
2	25	15	42.19	14.06	127.50	22.50	112.50	93.75	168.75	5.63
3	50	0	28.13	28.13	150.00	0.00	112.50	93.75	168.75	5.63
4	25	15	42.19	14.06	127.50	22.50	112.50	93.75	168.75	5.63
5	0	30	56.25	0.00	105.00	45.00	112.50	93.75	168.75	5.63
6	50	30	28.13	28.13	105.00	45.00	112.50	93.75	168.75	5.63
7	25	15	42.19	14.06	127.50	22.50	112.50	93.75	168.75	5.63
8	0	15	56.25	0.00	127.50	22.50	112.50	93.75	168.75	5.63
9	25	0	42.19	14.06	150.00	0.00	112.50	93.75	168.75	5.63
10	0	0	56.25	0.00	150.00	0.00	112.50	93.75	168.75	5.63
11	50	15	28.13	28.13	127.50	22.50	112.50	93.75	168.75	5.63
12	25	30	42.19	14.06	105.00	45.00	112.50	93.75	168.75	5.63
13	25	15	42.19	14.06	127.50	22.50	112.50	93.75	168.75	5.63

### 2.3 Kidney bean pod powder production

Kidney bean pods were dried in a tray dryer (Eksis, Turkey) at 50°C. The drying process ended when the moisture content of the pods fell below 10%. The dried kidney bean pods were ground using a blender (Waring, USA) and sieved through a sieve (500 μm). The resulting kidney bean pod powder was used as fat replacer and rice flour replacer in gluten‐free cake production.

### 2.4 Gluten‐free cake production

Gluten‐free cake production was implemented according to the formulation suggested by Gularte et al. (2012). The amount of oil and rice flour was differentiated according to kidney bean pod powder replacer ratio in the CCD design (Table 1). Three batches of cakes were prepared from each cake formulation. First, sugar and egg were mixed using hand type blender (Fakir, Ivy, Germany) for 1 min. Then milk, oil, baking powder, and kidney bean pod powder were mixed again for 3 min. Gluten‐free cake batter was poured on a cake mold (Karaca, Teemo, Turkey) and cooked in a preheated oven (Arçelik, SUF 5000, Turkey) at 170°C for 45 min. Gluten‐free cake was cooled at room temperature for 1 h, and then used for further analysis.

### 2.5 Moisture content

The moisture content of gluten‐free cake samples was analyzed according to method 44.40 recommended by AACC (2000).

### 2.6 Cooking loss

The weight of the gluten‐free cake batter and the weight of the baked gluten‐free cake were measured to calculate the cooking loss and the difference was determined. The percent of cooking loss value was determined by dividing the weight difference value by the weight of the cake batter (Dadalı, 2022).

### 2.7 Specific volume

A Volscan Profiler volume analyzer (Stable Microsystems, Surrey, UK) was used to determine the specific volume of gluten‐free cakes. While determining the specific volume value, gluten‐free cake volume was divided by gluten‐free cake weight and expressed as cm^3^/g.

### 2.8 Texture analysis

The modified method proposed by Dadalı and Elmacı (2019) was used to determine the texture profile of gluten‐free cakes. Gluten‐free cakes to be used in texture analysis were sliced at 2 cm thick. Gluten‐free cakes were analyzed with the TAXT Plus Texture Analyzer (Stable Micro Systems Ltd., Godalming, UK) using a 36‐mm‐diameter cylinder prop (P/36R) and a 5 kg load cell. A double compression test was applied and gluten‐free cakes were compressed to 50% of initial height at 5 mm/s test speed with a 5 s duration between cycles. The hardness, chewiness, and springiness values of gluten‐free cakes were calculated.

### 2.9 Color analysis

A hand‐type chromameter (Konica Minolta, CR 400, Japan) was utilized to define the color characteristics of gluten‐free cakes. Color measurement was performed from gluten‐free cake crumb and color characteristics were expressed as L* (brightness), a* [(−*a*): greenness, (+*a*): redness], and b* [(−*b*): blueness, (+*b*): yellowness].

### 2.10 Total phenolic content and antioxidant activity analysis

A total of 5 g of gluten‐free cake crumb was extracted with 80% (V/V) methanol for antioxidant activity and total phenolic content analysis. The extraction was implemented in a shaking water bath (Memmert, Germany) at 50°C for 90 min. The mixture was centrifuged at 3850 *g* for 15 min and the supernatant was diluted to 50 mL with 80% methanol. This sample extract solution was used for total phenolic content and antioxidant activity analysis.

Total phenolic content analysis was performed according to the modified method suggested by Dewanto et al. (2002). Fifty microliter gluten‐free cake extract was mixed with 3 mL distilled water and 250 μL of Folin–Ciocalteu reagent. Then, 750 μL of 7% Na_2_CO_3_ solution was added, and the mixture was allowed to stand for 8 min. At the end of 8 min, 950 μL of distilled water was added, and it was incubated for 2 h in the dark condition. The absorbance measurements were carried out at 765 nm with Agilent Cary 60 UV–Vis spectrophotometer (USA). Total phenolic content was expressed as gallic acid equivalent (GAE)/g through gallic acid calibration curve.

The antioxidant activity of gluten‐free cakes was evaluated using DPPH method (Bedoya et al., 2020). To determine antioxidant activity, 1950 μL of DPPH solution (100 μM) was added to 25, 50, and 75 μL of gluten‐free cake extract. Then, they were allowed to stand for 20 min at room conditions. Then, absorbance values of sample extracted were recorded at 517 nm using a spectrophotometer (Agilent, Cary 60, USA). The antioxidant activity results were expressed as μmol Trolox equivalent (TE)/g sample with the aid of % inhibition results of sample extracts and Trolox standard solutions.

### 2.11 Sensory analysis

The sensory evaluation of the gluten‐free cake samples was carried out with the participation of 52 panelists aged between 18 and 46 years. Panelists used a 9‐point hedonic scale (1: dislike extremely, 9: like very much) for evaluating gluten‐free cakes. Color, texture, flavor, and overall acceptability characteristics of gluten‐free cakes were evaluated by the panelists. Gluten‐free cakes were served as randomly coded with three‐digit numbers on white plastic plates and presented with water (Onoğur & Elmacı, 2015).

### 2.12 Composition of optimum gluten‐free cake formulation

Ash, fat, protein, and total dietary fiber content of optimum gluten‐free cake formulation and gluten‐free control cake were determined according to AOAC (2007) 923.03, 945.16, 968.06, and 991.43 numbered methods, respectively.

### 2.13 Statistical evaluation

Model fitting and optimization were implemented using Design Expert version 11.0. The evaluation of control and optimum gluten‐free cake was performed using analysis of variance (ANOVA) at the level of .05 (*n* = 3) by SPSS Statistics version 20.0.

## 3 RESULTS AND DISCUSSIONS

### 3.1 Model fitting

Response surface methodology (RSM) and CCD were used as optimization tools to determine optimum fat replacer‐to‐rice flour replacer ratio. Moisture content, cooking loss, specific volume, hardness, chewiness, springiness, L*, a*, b*, color, texture, flavor, and overall acceptability were independent variables and used as response values. The effects of fat and rice flour replacer ratios on response values are given in Table 2. The independent and dependent variables were fitted to the linear or quadratic model equation. While specific volume, hardness, chewiness, L*, antioxidant activity, texture, and overall acceptability responses were defined with quadratic model, moisture, cooking loss, springiness, a*, b*, total phenolic content, color, and flavor responses were defined with linear model. To determine linear, quadratic, and interaction effects of fat replacer and on the gluten‐free cake properties, ANOVA was implemented. The statistical significance of the model, factors, their interactions, and lack of fit are shown in Table 3. The calculated model value of each response was statistically significant at 95% confidence level (*p* > .05). The lack of fit shows the model failure of a model, and it should be insignificant (Varnalis et al., 2004). In this study, the lack‐of‐fit values of all responses were insignificant (*p* > .05). Gan et al. (2007) suggested that *R* ^2^ should be more than 80% for better model fitting. In agreement, *R* ^2^ values of all responses were higher than .85.

**TABLE 2:** Physical, chemical, and sensory properties of gluten‐free cakes produced according to experimental design.

Experiment no.	Moisture (%)	Cooking loss (%)	Specific volume (cm^3^/g)	Hardness (N)	Chewiness	Springiness	L*	a*	b*	Total phenolic content (mg GAE/g)	Antioxidant activity (μmol TE/g)	Color	Texture	Flavor	Overall acceptability
1	27.84 ± 0.53	7.42 ± 0.14	2.09 ± 0.04	19.21 ± 0.36	0.66 ± 0.01	0.29 ± 0.01	61.86 ± 1.18	3.22 ± 0.16	26.16 ± 0.55	0.51 ± 0.05	1.78 ± 0.15	8.21 ± 0.82	8.79 ± 0.68	7.7 ± 0.56	8.13 ± 0.81
2	27.52 ± 0.39	7.39 ± 0.10	2.12 ± 0.03	18.74 ± 0.56	0.66 ± 0.01	0.25 ± 0.00	61.91 ± 0.87	3.17 ± 0.04	25.98 ± 0.36	0.55 ± 0.04	1.85 ± 0.16	8.25 ± 0.75	8.75 ± 0.80	7.75 ± 0.71	8.45 ± 0.77
3	26.96 ± 0.43	7.57 ± 0.12	2.14 ± 0.03	16.24 ± 0.22	0.64 ± 0.04	0.28 ± 0.00	62.38 ± 1.00	2.56 ± 0.04	25.54 ± 0.41	0.46 ± 0.01	1.63 ± 0.03	8.23 ± 0.13	8.48 ± 0.14	7.75 ± 0.55	8.37 ± 0.75
4	28.01 ± 0.87	7.28 ± 0.23	2.09 ± 0.06	19.18 ± 0.59	0.62 ± 0.02	0.26 ± 0.01	62.25 ± 1.03	3.45 ± 0.11	26.98 ± 0.84	0.53 ± 0.02	1.79 ± 0.06	8.40 ± 0.26	8.19 ± 0.25	7.59 ± 0.24	8.07 ± 0.25
5	28.92 ± 0.54	7.22 ± 0.12	1.97 ± 0.02	25.53 ± 0.76	0.78 ± 0.02	0.22 ± 0.00	61.72 ± 1.03	3.95 ± 0.09	27.54 ± 0.57	0.62 ± 0.02	1.96 ± 0.05	8.39 ± 0.69	6.85 ± 0.46	7.89 ± 0.62	7.45 ± 0.41
6	29.98 ± 0.25	6.85 ± 0.07	1.25 ± 0.02	42.25 ± 1.16	0.95 ± 0.03	0.16 ± 0.00	57.49 ± 0.56	5.27 ± 0.03	31.78 ± 0.24	0.93 ± 0.07	2.78 ± 0.18	8.59 ± 0.41	5.43 ± 0.73	8.15 ± 0.69	5.05 ± 0.65
7	27.83 ± 0.19	7.37 ± 0.05	2.13 ± 0.02	17.43 ± 0.69	0.68 ± 0.00	0.29 ± 0.03	61.75 ± 0.44	2.98 ± 0.12	26.78 ± 0.18	0.53 ± 0.01	1.84 ± 0.11	8.35 ± 0.57	8.12 ± 0.76	7.63 ± 0.54	8.29 ± 0.15
8	26.85 ± 0.06	7.61 ± 0.02	2.17 ± 0.00	12.87 ± 1.02	0.62 ± 0.00	0.29 ± 0.01	62.75 ± 0.74	1.94 ± 0.03	25.18 ± 0.79	0.42 ± 0.03	1.57 ± 0.03	8.17 ± 0.71	8.27 ± 0.51	7.67 ± 0.73	8.59 ± 0.44
9	26.73 ± 0.29	7.63 ± 0.08	2.25 ± 0.03	10.57 ± 0.87	0.60 ± 0.03	0.31 ± 0.01	63.04 ± 0.70	1.35 ± 0.08	24.82 ± 0.22	0.38 ± 0.01	1.42 ± 0.01	8.03 ± 0.53	8.14 ± 0.65	7.45 ± 0.47	8.42 ± 0.84
10	26.56 ± 0.34	7.66 ± 0.12	2.28 ± 0.03	9.09 ± 0.52	0.54 ± 0.01	0.32 ± 0.02	63.48 ± 1.02	0.72 ± 0.07	20.27 ± 0.50	0.14 ± 0.01	1.06 ± 0.04	7.98 ± 0.44	8.08 ± 0.69	7.20 ± 0.53	7.67 ± 0.62
11	29.05 ± 0,26	7.06 ± 0.06	1.78 ± 0.01	30.47 ± 0.29	0.80 ± 0.08	0.20 ± 0.02	59.73 ± 0.52	4.37 ± 0.24	29.56 ± 0.27	0.69 ± 0.08	2.12 ± 0.26	8.44 ± 0.33	6.45 ± 0.31	7.95 ± 0.71	6.73 ± 0.56
12	29.37 ± 0.36	6.92 ± 0.10	1.67 ± 0.03	33.12 ± 0.62	0.89 ± 0.01	0.18 ± 0.00	59.23 ± 0.81	4.89 ± 0.08	30.2 ± 0.34	0.76 ± 0.04	2.34 ± 0.17	8.52 ± 0.67	6.31 ± 0.44	8.08 ± 0.55	6.32 ± 0.31
13	27.80 ± 0.17	7.35 ± 0.14	2.12 ± 0.02	17.27 ± 0.81	0.65 ± 0.05	0.25 ± 0.03	62.17 ± 0.96	2.85 ± 0.12	26.53 ± 0.87	0.51 ± 0.04	1.82 ± 0.09	8.27 ± 0.62	8.27 ± 0.76	7.65 ± 0.39	8.38 ± 0.49

*Note*: The results are given as arithmetic mean ± standard deviation.

**TABLE 3:** ANOVA results of response variables (*X*_1_: fat replacer, *X*_2_: rice flour replacer).

	Moisture	Cooking loss	Specific volume	Hardness	Chewiness	Springiness	L*	a*	b*	Total phenolic content	Antioxidant activity	Color	Texture	Flavor	Overall acceptability
Model	<0.01	<0.01	<0.01	<0.01	<0.01	<0.01	<0.01	<0.01	<0.01	<0.01	<0.01	<0.01	<0.01	<0.01	<0.01
*X*_1_	<0.01	<0.01	<0.01	<0.01	<0.01	<0.01	<0.01	<0.01	<0.01	<0.01	<0.01	<0.01	0.06	<0.01	<0.01
*X*_2_	<0.01	<0.01	<0.01	<0.01	<0.01	<0.01	<0.01	<0.01	<0.01	<0.01	<0.01	<0.01	<0.01	<0.01	<0.01
*X*_1_ *X*_2_			<0.01	0.02	0.29		<0.01				0.04		0.11		<0.01
$X_{1}^{2}$			<0.01	0.04	0.30		0.16				0.89		0.11		0.04
$X_{2}^{2}$			<0.01	0.03	0.02		0.08				0.22		0.01		0.01
Lack of fit	0.06	0.09	0.07	0.08	0.15	0.55	0.08	0.42	0.10	0.10	0.09	0.97	0.09	0.16	0.06
*R* ^2^	0.91	0.90	0.99	0.98	0.96	0.87	0.98	0.97	0.95	0.98	0.99	0.92	0.87	0.89	0.96

### 3.2 Moisture content and cooking loss

**FIGURE 1:**
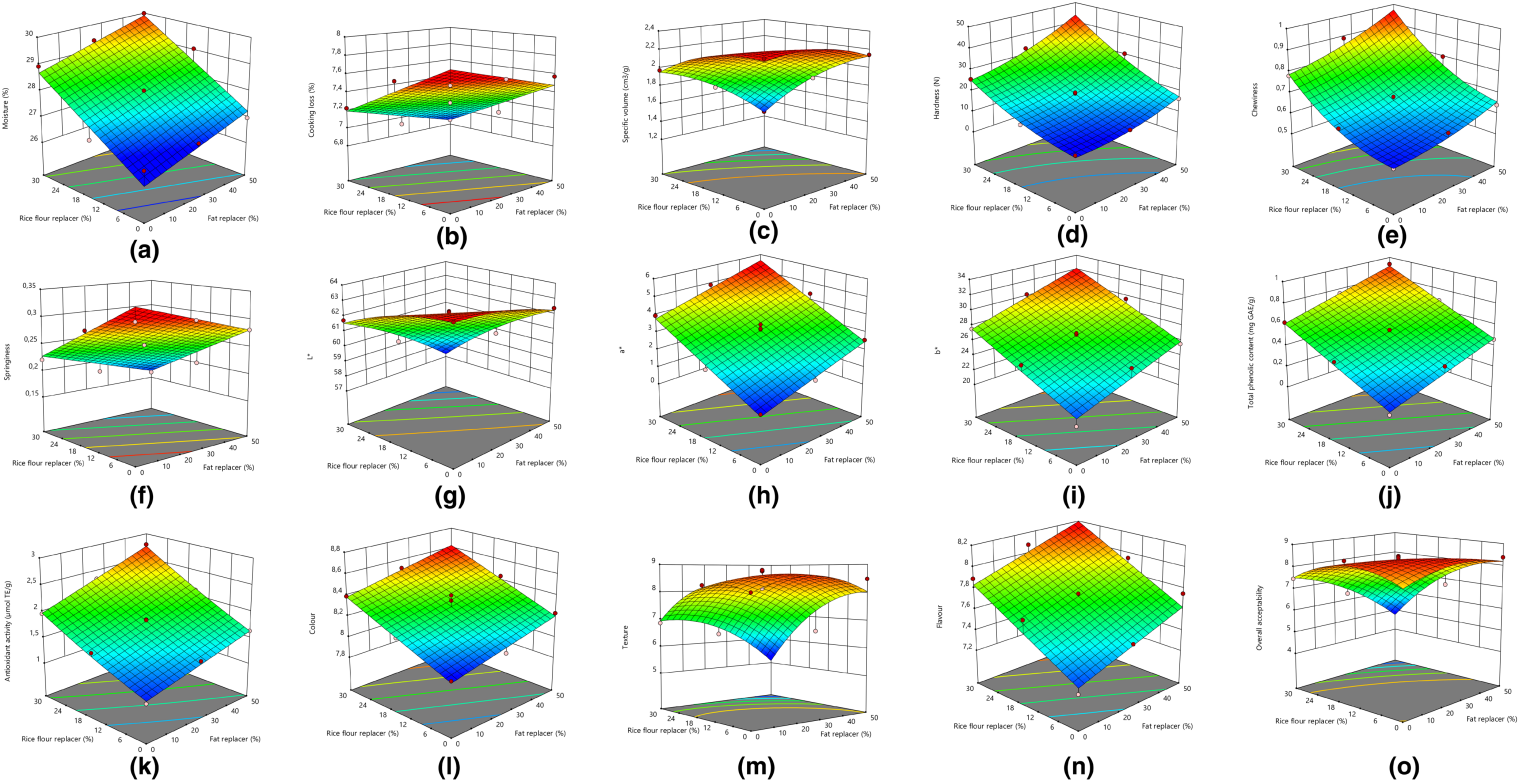
The calculated effect of fat and rice flour replacer on moisture (a), cooking loss (b), specific volume (c), hardness (d), chewiness (e), springiness (f), L* (g), a* (h), b* (i), total phenolic content (j), antioxidant activity (k), color (l), texture (m), flavor (n), and overall acceptability (o).

Moisture content and cooking loss responses were defined with linear model. Fat and rice flour replacer affected the moisture content of gluten‐free cakes linearly (*p* < .05). The moisture content of gluten‐free cakes increased with the increase in fat and rice flour replacer used in gluten‐free cake formulation (Figure 1a). The gluten‐free cake with 50% fat replacer and 30% rice flour replacer had the highest moisture content (29.98%) (Table 2). Cooking loss value was linearly affected by fat and rice flour replacer. The use of fat and rice flour replacer negatively affected cooking loss (Figure 1b). Cooking loss values of gluten‐free cakes are in the range of 6.85% (exp. 6)–7.66% (exp. 10) (Table 2). Due to the fact that dietary fiber retains water during the cooking process, the cooking loss was less and the moisture content of the gluten‐free cakes containing kidney bean pod powder was higher. Similar to this study, Brigagão et al. (2021) found higher moisture content in gluten‐free muffins enriched with pineapple peel‐ and banana peel‐containing high dietary fiber.

### 3.3 Specific volume and textural properties

Fat and rice flour replacer affected the specific volume of gluten‐free cakes linearly and quadratically (*p* < .05). The use of fat and rice flour replacer in gluten‐free cakes resulted in a reduction in specific volume (Figure 1c). The specific volume of the gluten‐free control cake without replacer was the highest (2.28 cm^3^/g) and very close to the gluten‐free cake with 25% fat replacer (2.25 cm^3^/g) (Table 2). Consistent with this study, Saeidi et al. (2018) found that the gluten‐free cake was enriched with pomegranate seeds and had lower specific volume. The specific volume of gluten‐free muffins was decreased by the inclusion of black carrot fiber in gluten‐free muffin formulation. The decrease in specific volume of gluten‐free muffins was explained by the fact that dietary fiber causes the collapse of carbon dioxide gas bubbles during baking (Singh et al., 2016).

Fat and rice flour replacers had positive linear and quadratic effects on the hardness value of gluten‐free cakes (*p* < .05). Chewiness value was affected linearly by fat and flour replacer and quadratically by rice flour replacer (*p* < .05). Fat and rice flour replacers had linear effects on the springiness values of gluten‐free cakes (*p* < .05). The hardness and chewiness values of the gluten‐free cake containing 50% fat replacer and 30% rice flour replacer were higher than the other samples (Figure 1d,e). However, the springiness value (0.16) was lower than the other samples. It was thought that kidney bean pod powder decreased the amount of gas held by dough thorough thickening of walls surrounding the gas cells, which resulted in higher gluten‐free cake hardness (Nouri et al., 2017). Springiness is related to the strength of the crumb cell network and chewiness is characterized as the energy needed to break into food and prepare it for swallowing (Azari et al., 2020; Belghith‐Fendri et al., 2016). The use of kidney bean pod powder as a fat and rice flour replacer in the gluten‐free cake formulation increased the hardness and chewiness value while decreasing the springiness value (Figure 1f). Similar to this study, gluten‐free cakes with apple, orange, and carrot pomace showed an increase in hardness and a decrease in springiness (Kırbaş et al., 2019). In addition, it was observed that the hardness and chewiness values of the gluten‐free cakes increased with the use of unripe banana peel in gluten‐free cake (Türker & Savlak, 2021).

### 3.4 Color properties

The use of kidney bean pod powder as a fat and rice flour replacer in gluten‐free cake had linear effects on the L* value. In addition, there was an interaction effect on the L* value of fat and rice flour replacer. The brightest gluten‐free cake was the gluten‐free cake without fat and rice flour replacer having 63.48 L* value. Fat and rice flour replacer used in gluten‐free cake reduced brightness value of the gluten‐free cake (Figure 1g). There were linear effects of fat and rice flour replacer on both a* and b* values. It was determined that redness and yellowness of gluten‐free cakes increased with the increase in the ratio of fat and rice flour replacer (Figure 1h,i). The a* and b* values of gluten‐free cakes were in the range of 0.72–5.27 and 20.27–31.78, respectively. As expected, the use of kidney bean pod powder instead of fat and rice flour influenced the color characteristics of gluten‐free cake. Similar to this study, the use of apple pomace in gluten‐free cake decreased L* value and increased a* and b* values (Azari et al., 2020).

### 3.5 Total phenolic content and antioxidant activity

Total phenolic content of gluten‐free cakes was linearly affected by both fat and rice flour replacer (*p* < .05). Fat and rice flour replacer have both linear effects and interaction effects on the antioxidant activity (*p* < .05). The total phenolic content and antioxidant activity values increased with the use of kidney bean pod powder as fat and rice flour replacer in gluten‐free cake (Figure 1j,k). Gluten‐free cake containing 50% fat and 30% rice flour replacer has the highest total phenolic content (0.93 mg GAE/g) and antioxidant activity value (2.78 μmol TE/g) (Table 2). In accordance with this study gluten‐free crackers enriched with green tea leaves showed significant total phenolic content and antioxidant activity increase (Radočaj et al., 2014). In addition, cake with artichoke bracts had higher total phenolic content and antioxidant activity than cake without artichoke bracts (Dadalı, 2022). When broccoli leaves were used for gluten‐free cake fortification, gluten‐free cakes represented higher total phenolic content and antioxidant activity (Drabińska et al., 2018).

### 3.6 Sensory properties

Gluten‐free cakes were evaluated for color, texture, flavor, and overall acceptability. Color, flavor, texture, and overall acceptability properties of gluten‐free cakes containing kidney bean pod powder were linearly affected by fat and rice flour replacer (*p* < .05). Rice flour replacer had a quadratic effect on the texture properties of gluten‐free cakes (*p* < .05). In addition, overall acceptability was influenced by interaction effect of fat and rice flour replacer, the quadratic effect of the fat replacer, and the quadratic effect of the rice flour replacer (*p* < .05). The color characteristics of the gluten‐free cakes were liked more by the panelists as the fat and rice flour replacer ratio increased (Figure 1l). The texture of gluten‐free cakes having 50% fat replacer (exp 3), 25% fat replacer and 15% rice flour replacer (exp 4), 15% rice flour replacer (exp 8), and 25% fat replacer (exp 9) were more liked than control gluten‐free cake (Figure 1m). The flavor of the gluten‐free cakes was liked more with the increase in the use of kidney bean pod powder as fat and rice flour replacer in gluten‐free cakes (Table 2) (Figure 1n). The use of kidney bean pod powder had a positive effect on overall acceptability of gluten‐free cakes except for gluten‐free cake with 30% rice flour replacer (exp 5), 50% fat and 30% rice flour replacer (exp 6), 50% fat and 15% rice flour replacer (exp 11), 25% fat and 30% rice flour replacer (exp 12), and 25% fat and 15% rice flour replacer (exp 13) (Figure 1o). Consistent with this study, the use of high amounts of broccoli leaves in gluten‐free cakes decreased overall acceptance scores (Krupa‐Kozak et al., 2019).

### 3.7 Optimization

Numerical optimization approach was used to determine the optimum gluten‐free cake formulation enriched with kidney bean pod powder. Optimization was implemented using Design Expert version 11.0 software for simultaneous optimization of multiple responses, namely moisture content, cooking loss, specific volume, hardness, chewiness, springiness, color values, and sensory analysis results. As a result of optimization, it was determined that optimum use of kidney bean pod powder as fat replacer was 27.88% and rice flour was 13.52%. The verification experiments were implemented for optimum gluten‐free cake formulation and the results were given in Table 4. The percent error value of the experimental data for the physical, chemical, and sensory properties of the gluten‐free cake estimated from the model was between 1.09 and 8.34%. The predicted results from the model were in agreement with the experimental analysis results and there was no statistically significant difference between them (*p* < .05).

**TABLE 4:** The verification experiment results.

	Predicted value	Experimental value^a^	SE	Difference	% Error^b^	*p* Value
Moisture (%)	27.89	28.2 ± 0.54	0.27	−0.31	1.09	.31
Cooking loss (%)	7.34	7.01 ± 0.31	0.16	0.33	4.77	.10
Specific volume	2.10	2.03 ± 0.06	0.03	0.07	3.59	.08
Hardness (N)	18.38	19.34 ± 1.03	0.51	−0.96	4.96	.13
Chewiness	0.66	0.65 ± 0.01	0.00	0.01	1.47	.10
Springiness	0.26	0.24 ± 0.02	0.01	0.02	6.65	.12
L*	61.88	60.05 ± 1.92	0.96	1.83	3.05	.13
a*	3.08	2.90 ± 0.24	0.12	0.18	6.34	.21
b*	26.67	25.54 ± 2.23	1.11	1.13	4.43	.19
Total phenolic content (mg GAE/g)	0.54	0.51 ± 0.04	0.02	0.03	5.13	.28
Antioxidant activity (μmol TE/g)	1.81	1.93 ± 0.15	0.25	−0.12	6.16	.00
Color	8.29	8.07 ± 0.49	0.19	0.22	2.70	.36
Texture	8.32	8.01 ± 0.46	0.23	0.31	3.82	.25
Flavor	7.72	7.36 ± 0.44	0.22	0.36	4.90	.18
Overall acceptability	8.23	7.60 ± 0.77	0.38	0.63	8.34	.18

Abbreviation: SE, mean standard error.

^a^ The results were given as arithmetic mean ± standard deviation.

^b^ % Error = (|*y*_exp_ − *y*_pre_|/*y*_exp_) × 100.

The comparison of the composition results of the gluten‐free cake containing the optimum amount of fat and rice flour with the gluten‐free control cake is given in Table 5. Gluten‐free cakes containing optimum amount of kidney bean pod powder had higher moisture, ash, protein, and total dietary fiber than control gluten‐free cake, while fat content of optimum gluten‐free cake was lower than control gluten‐free cake (*p* < .05). The total dietary fiber content of the gluten‐free cake formulation containing the optimum amount of kidney bean pod powder is approximately four times higher than the gluten‐free control cake. Codex Alimentarius Commission (2007) stated that solid foods can be classified as source fiber containing higher than 3 g/100 g total dietary fiber. According to the Codex Alimentarius Commission (2007), optimum gluten‐free cake containing kidney bean pod powder as fat replacer 27.88% and rice flour 13.52% can be classified as fiber source.

**TABLE 5:** Chemical composition of control and optimum gluten‐free cakes.

	Control gluten‐free cake	Optimum gluten‐free cake
Moisture (%)	26.56 ± 0.34^a^	28.2 ± 0.54^b^
Ash (%)	1.19 ± 0.11^a^	1.81 ± 0.03^b^
Fat (%)	12.26 ± 0.35^b^	9.33 ± 0.23^a^
Protein (%)	4.64 ± 0.08^a^	5.89 ± 0.11^b^
Total dietary fiber (%)	1.13 ± 0.09^a^	4.43 ± 0.18^b^

*Note*: The results were given as arithmetic mean ± standard deviation. Means along a row with different superscripts are significantly different (*p* < .05).

## 4 CONCLUSION

The optimum usage ratio of kidney bean pod powder as fat and rice flour replacer in gluten‐free cake was investigated and it was determined that kidney bean pod powder can be used as 27.88% fat and 13.52% rice flour replacer under optimum conditions. Gluten‐free cake containing kidney bean pod powder as a fat and rice flour replacer can be a new alternative with improved nutritional and sensory properties for people on a gluten‐free diet and celiac patients. The gluten‐free cake rich in protein, phenolic compounds, antioxidant activity, and dietary fiber with improved sensory properties can be offered to consumers who consume a gluten‐free cake containing the optimum amount of kidney bean pod powder. By using kidney bean pod powder in gluten‐free cake, vegetable production by‐products were valorized, added value was gained, sustainability was contributed, and a healthier alternative product was obtained for people on a gluten‐free diet and celiac patients. The optimum gluten‐free cake with kidney bean pod powder is a fiber source and nutritive new gluten‐free product. This optimum gluten‐free cake formulation will guide the food industry in producing gluten‐free products. In the following studies, kidney bean pod powder can be used in different bakery products to improve the physical, chemical, and sensory properties of these products.

## AUTHOR CONTRIBUTIONS

**Ceyda Dadalı:** Conceptualization (lead); data curation (lead); investigation (lead); methodology (lead); resources (lead); writing – original draft (lead); writing – review and editing (lead).

## CONFLICT OF INTEREST STATEMENT

The author declares no conflict of interest.

## Data Availability

The data that support the findings of this study are available on request from the corresponding author.
